# Fetal Wilm's tumor detection preceding the development of isolated lateralized overgrowth of the limb: a case report and review of literature

**DOI:** 10.3389/fped.2024.1334544

**Published:** 2024-03-18

**Authors:** Elie Bechara, Chloé Saadé, Caroline Geagea, Daniel Charouf, Pauline Abou Jaoude

**Affiliations:** ^1^ Department of Pediatrics and Adolescent Medicine, Faculty of Medicine, American University of Beirut, Beirut, Lebanon; ^2^ Children Cancer Institute, American University of Beirut Medical Center, Beirut, Lebanon; ^3^ Department of Pediatrics, Hôtel-Dieu de France, University Medical Center, Faculty of Medicine, Saint-Joseph University of Beirut, Beirut, Lebanon; ^4^ Department of Pediatrics, Bellevue Medical Center, Mansourieh, Lebanon; ^5^ Division of Pediatric Nephrology, Hôtel-Dieu de France, University Medical Center, Faculty of Medicine, Saint-Joseph University of Beirut, Beirut, Lebanon

**Keywords:** fetal Wilms tumor, prenatal diagnosis, isolated lateralized overgrowth, hemihypertrophy, Beckwith–Wiedemann syndrome, cancer, polyhydramnios

## Abstract

Fetal Wilms tumor (WT) is extremely rare, but with advances in fetal imaging, more cases are being reported. The management of these cases remains challenging. Herein, we present the case of a full-term female infant diagnosed antenatally at 32 weeks of gestation with a right solid renal mass detected on routine prenatal ultrasound without polyhydramnios. At birth, the infant was healthy, with no evidence of dysmorphic features or abnormal laboratory tests to suggest a predisposition syndrome. Her family history was also unremarkable. A successful radical right nephrectomy was performed on day 2 of life revealing a classic WT. She received vincristine as adjuvant chemotherapy without any complications. At the age of 1 month, the infant developed isolated lateralized overgrowth of the right lower limb suspicious of Beckwith–Wiedemann syndrome. At the latest follow-up of 4 years, the child is healthy and disease-free with conserved asymmetry of lower limbs. The case provides insights into the challenging diagnosis and treatment of fetal WT. A review of the literature suggests that the presence of polyhydramnios is a worse prognostic factor while the combination of best supportive care and surgery remains the best management. Fetal WT can be associated with predisposition syndromes; however, their first manifestations can develop after the diagnosis of cancer has been made, as in our patient. We propose starting active surveillance programs and genetic testing for any case of fetal WT.

## Introduction

Fetal tumors are rare entities with an estimated prevalence of 7–10 per 100,000 live births. Only 5% of these tumors arise from the kidneys (1, 2). Wilms tumor (WT) is the second most common neonatal renal tumor after congenital mesoblastic nephroma (CMN), accounting for approximately 20% of all cases, with 16% of them identified prenatally as an abdominal mass (3). Some cases are associated with prenatal polyhydramnios and fetal hydrops (4). Less than 10% of neonatal WT cases are associated with congenital syndromes such as Wilms tumor, aniridia, genitourinary abnormalities, range of developmental delays (WAGR) syndrome, Beckwith–Wiedemann syndrome (BWS)/isolated lateralized overgrowth (ILO), Denys–Drash syndrome, and Simpson–Golabi–Behmel syndrome (2, 5). In the present study, we report the case of a fetal WT detected in the third trimester by routine prenatal ultrasound in a female infant before she developed isolated hemihypertrophy of her lower limb postnatally.

## Methods

A PubMed search was performed for articles indexed through the Medline database. Keywords used included “fetal renal tumors” and “Wilms Tumor” or “Wilms’ Tumour” and “prenatal diagnosis”. In total, 44 articles were identified, of which 13 were case reports of interest. A total of 13 different patients were described within these 13 case reports published between 1984 and 2021 (Table 1). A total of 31 articles were excluded: 16 articles were not about WT, 13 were not related, and 2 were not case reports. Furthermore, when we added the term “hemihypertrophy,” we retrieved only one article that was not related to our topic. A qualitative analysis was performed on the included cases, and the findings are presented.

**Table 1:** Reported cases of antenatal Wilms tumor.

Article	Diagnosis	Delivery	Surgery	Stage	Treatment	Outcome
Roth (6)	Third trimester, Bilateral renal lesions, teratoma, nephroblastomatosis	NA	Radical right, partial left nephrectomy at day 10 of life	V	NA	Alive, tumor-free at 3-year follow-up
Tomá et al. (7)	37 WG, Left renal mass	Full term	Left nephrectomy at day 12 of life	I	None	NA
Suresh et al. (8)	37 WG, Left renal mass, polyhydramnios	CS at 37 WG for loss of fetal movements	Autopsy	I	None	Stillbirth
Applegate et al. (9)	36 WG, right renal mass	Full-term	Right nephrectomy	I	None	Alive
Vadeyar et al. (10)	28 WG, left renal mass, polyhydramnios, hydrops fetalis	CS at 28 WG for fetal distress	Autopsy	I	None	Deceased at day 1 of life
Linam et al. (11)	33 WG, right renal mass, nodules on left kidney	CS at 38 WG (elective)	Right Nephrectomy at day 3 of life	V	EE4A	Alive, tumor-free at 1 year follow-up
Jain et al. (12)	7 months of gestation, left renal mass	Full-term	Resection at 7 months of age with intraoperative spill	III	DD4A-ICE	Deceased
Sarin et al. (13)	29 WG, left renal mass, polyhydramnios	CS at 34 WG for hydrops fetalis and fetal distress	Nephrectomy at day 4 of life	I	None	Deceased at day 2 post-surgery
Mitra et al. (14)	34 WG, bilateral renal mass, oligoamnios, ductal plate malformation, polysplenia, multiple skeletal malformations, pulmonary hypoplasia	NVD at 41 WG	Autopsy	V	None	Deceased at day 1 of life
Toussi et al. (15)	32 WG, left renal mass	NA	Laparoscopic nephrectomy at day 26 of life	II	EE4A	Alive, tumor-free at 6-month follow-up
Ogawa et al. (16)	29 WG, left renal mass	CS at 34 WG for fetal distress	Nephrectomy at day 4 of life	III	DD4A	Alive, tumor-free at 3-month follow-up
Rampersad et al. (17)	34 WG, left renal mass; polyhydramnios	CS at 35 WG for spontaneous rupture of membranes	Radical nephrectomy at 17 days of age	I	None	Deceased at 10 days post-surgery
Meng et al. (18)	38 WG, bilateral renal mass	NA	NSS separately for each side at 1 and 3 months of age	V	VA/CDCV	Alive, tumor-free at 1 year follow-up

NA, not available, EE4A, vincristine and dactinomycin regimen; DD4A, vincristine; dactinomycin and doxorubicin; ICE, ifosphomide; carboplatin and etoposide regimen; VA, vincristine; actinomycin; CDCV, carboplatin, doxorubicin, cyclophosphamide, VP16; NSS, nephron-sparing surgery; NVD, normal vaginal delivery.

## Case report

**Figure 1:**
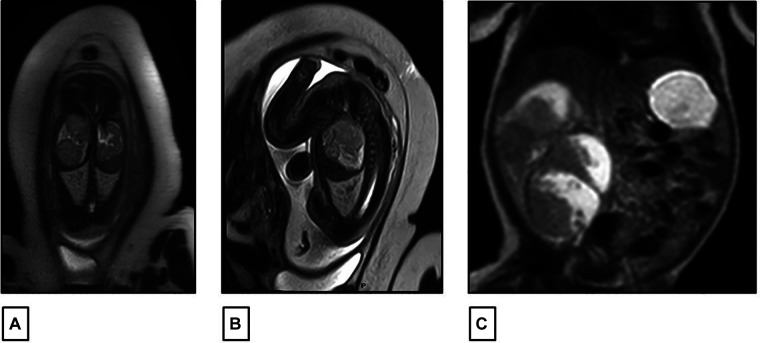
(**A,B**) Fetal MRI performed at 34 weeks of gestation showing a right renal fossa occupied by a 60 mm × 30 mm multinodular solid mass with intermediate signal intensity on T2. (**C**) Postnatal abdominal MRI showing a lobulated heterogeneous mass in the right renal fossa 81 mm × 58 mm.

A 27-year-old previously healthy primigravida mother with an uneventful pregnancy presented at 32 weeks of gestation (WG), with a routine prenatal ultrasound showing an isolated heterogeneous fetal mass of 2 cm occupying the right renal fossa. Family history was negative for any malignancies or renal diseases. At 34 WG, a fetal magnetic resonance imaging (MRI) scan was performed and confirmed the presence of a solid multinodular right renal mass of 6 cm with a normal contralateral kidney and no other associated congenital malformations (Figures 1A,B). The pregnancy went smoothly until term, with weekly follow-up fetal ultrasound examinations showing a gradual increase in the size of the mass but with normal amniotic fluid and fetal growth. There were no signs of fetal distress. The delivery was performed at 38 WG by elective cesarean section (CS) to avoid mass rupture.

At birth, the female infant was healthy with a good Apgar score. The examination of the placenta was normal and did not show placental mesenchymal dysplasia. The infant’s physical exam was unremarkable except for a palpable right upper quadrant mass. No macroglossia, omphalocele, macrosomia, or other dysmorphic features were detected. Laboratory workups were carried out and were normal, except for a mild elevation of serum lactate dehydrogenase (LDH) at 534 U/L. There were no recorded episodes of hypoglycemia or hypercalcemia. An abdominal ultrasound was also controlled postnatally, revealing a significant increase in tumor size to 8 cm. The mass had multiple lobules surrounded by vessels without a clear intra-tumoral Doppler flow. The left kidney appeared normal in size and echogenicity with no dilatation of the excretory system. The liver and spleen were normal; there was no evidence of nephrogenic rest, nor adrenal cytomegaly or pancreatic adenomatosis. There was no evidence of renal vein or inferior vena cava thrombus on Doppler study. A complementary abdominal MRI scan was also performed on the same day, confirming the same radiological findings with a lobulated heterogeneous mass at the right renal fossa, measuring 81 mm × 58 mm, with hyper- and hypo-intense signals respectively on T1 and T2. It was well limited to the kidney with no evidence of enlarged lymph nodes (Figure 1C).

**Figure 2:**
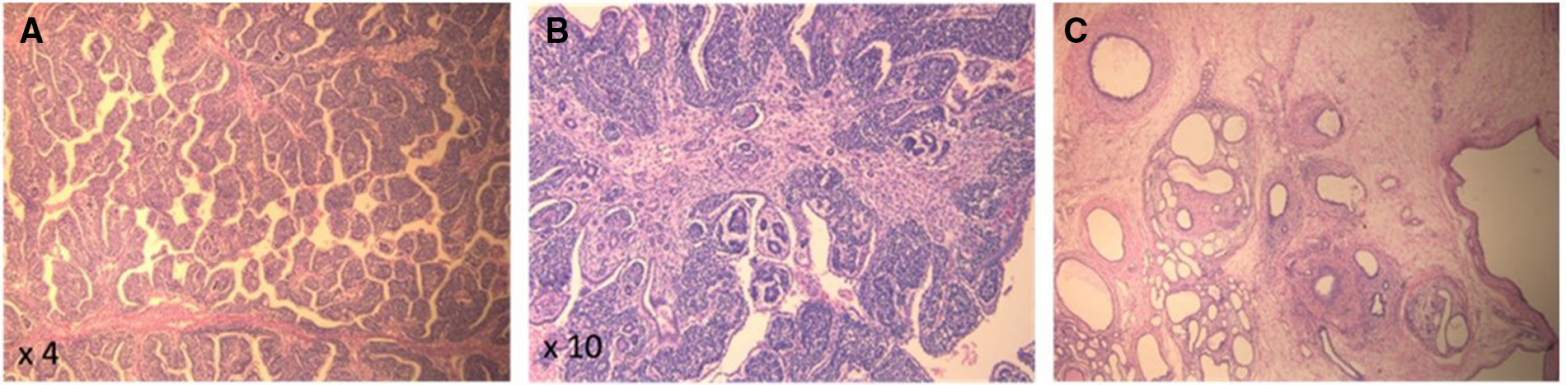
(**A,B**) Pathology (H&E) of the kidney tumor showing pupillary structures supported by triphasic neoplastic proliferation comprising immature glomerular and tubular structures and immature small round blue cells. (**C**) Background renal parenchyma showing microcystic dilated tubules, strangled by concentric layers of spindled mesenchymal cells.

On the second day of life, the patient underwent a right radical nephrectomy with excision of the supra-hilar and para-aortic lymph nodes. A pathology study revealed a well-circumscribed and encapsulated tumoral proliferation that was limited to the kidney (Figure 2). It contained three types of cells, composed of epithelial cells that formed immature tubules and primitive-looking glomeruli, mesenchymatous cells made up of spindle cells, and blastemal cells that were round and blue with scanty cytoplasm. Foci of necrosis were present. The adjacent renal parenchyma showed cystic dilations in the cortex and medulla. The cysts were lined by a flattened cuboid epithelium. No anaplastic features were identified. There was no vascular invasion, and the excised lymph nodes had no signs of malignancy. Thus, the diagnosis of triphasic WT or nephroblastoma was confirmed. Staging was completed with a computed tomography scan of the chest, which came back normal, and the tumor was classified as stage I according to the International Society of Pediatric Oncology (SIOP) and the National Wilms Tumor Study (NWTS) Group Staging System (19). Three weeks after surgery, adjuvant chemotherapy was started with weekly vincristine for a total of 10 weeks, without major adverse events.

**Figure 3:**
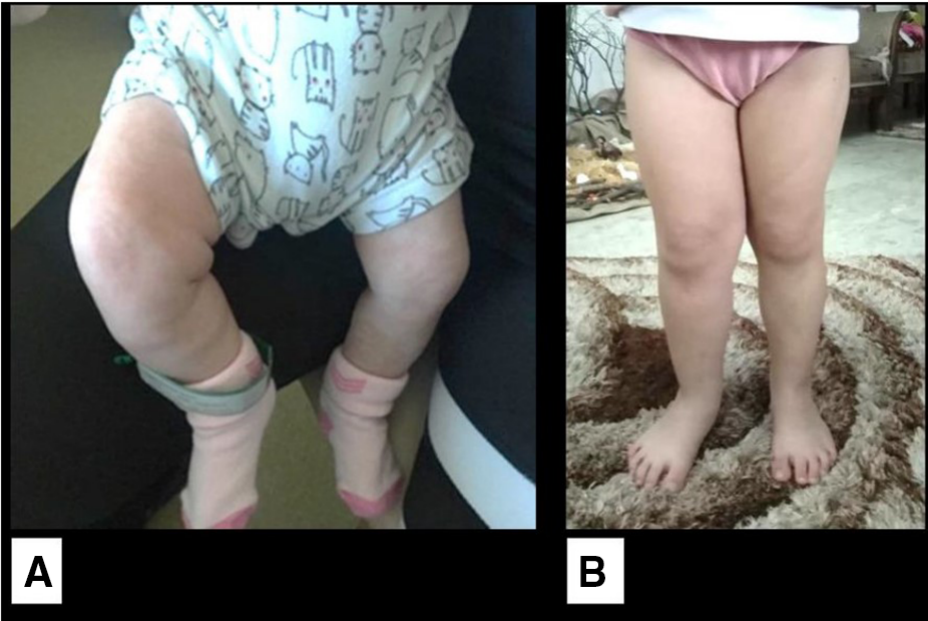
Isolated lateralized hemihypertrophy of the right lower limb. (**A**) At 1 month of age. (**B**) At 4 years of age.

At 1 month of age, the infant developed an isolated right lower limb hemihypertrophy without skin staining (Figure 3). No vascular or capillary limb malformation was noted on Doppler study. The cardiac ultrasound and retinal examination were unremarkable. With the addition of this new clinical feature, Beckwith–Wiedemann syndrome was highly suspected and genetic testing was recommended. However, the parents did not give their consent to proceed with molecular studies. The child had since then regular clinical, biological, and radiological surveillance every 3 months. At the last follow-up at 4 years of age, the girl had normal growth (75th percentile for weight and height) and developmental milestones for her age, with no tumor recurrence and stable lower limb asymmetry with a difference of 1 cm.

## Discussion

Fetal renal tumors are defined as tumors involving the kidney discovered before birth. They are extremely rare, occurring in 7 out of 100,000 births (20). The majority are benign, with CMN being the most common tumor. WT, on the other hand, is the most common malignant renal tumor, with a prevalence of 0.12% (21). Reports of fetal WT are scarce and are limited to case reports (Table 1).

In their review, Berger and von Schweinitz (1) proposed an approach to the management of fetal kidney tumors. Prenatal ultrasound remains the most preferred modality in the diagnosis and follow-up of antenatal renal tumors. They are usually detected in the third trimester and have a predilection to the right side. Although prenatal fetal MRI does not differentiate between CMN and WT (22), it is highly valuable in evaluating large renal masses (>10 cm), in assessing the contralateral kidney and adjacent structures and in planning resection. Since the majority of fetal tumors diagnosed early are either benign or low-stage malignant tumors, Berger and von Schweinitz recommended that initial management should focus on immediate counseling and perinatal care. When it comes to delivery, vaginal deliveries can be safely performed in non-complicated cases, while a cesarean section is indicated for complicated pregnancies and large renal tumors (23). Postnatal management of prenatally detected renal tumors remains surgical (1). There is no indication for percutaneous needle biopsy unless there is evidence of metastatic disease or unresectable tumor (24). Once the newborn has achieved adequate postnatal adaptation and hemodynamic stability, a radical nephrectomy should be attempted. Complete surgical resection with negative margins and without tumoral spillage is recommended to prevent local recurrence. Although nephron-sparing surgery is gaining in popularity (25), it is still reserved for cases of either bilateral WT or whenever there is concern for contralateral kidney involvement or function (26).

Due to the low incidence of fetal WT, management has not been standardized, but it is in line with what has been described for fetal kidney tumors in general. In our review, all 13 cases were diagnosed in the third trimester using routine ultrasound. There were four bilateral WTs, seven tumors originating from the left kidney, and two from the right kidney. Five cases were associated with polyhydramnios. There were five preterm deliveries secondary to fetal distress. Delivery was by CS in six patients. Radical nephrectomy was performed in eight patients while three patients with bilateral WT had nephron-sparing surgery. Tumor staging was low: six tumors were stage 1; and one tumor was stage 2. Only two tumors were stage 3. Five patients received adjuvant chemotherapy. None of the reviewed cases were associated with BWS or ILO; however, there was one case of nephroblastomatosis (6), which is a condition generally associated with WT.

In general, the outcome of WT is excellent, with overall survival (OS) of 90% at 5 years (24, 27). In their report, van den Heuvel-Eibrink et al. showed that patients with WT aged less than 7 months had an OS of 93%; however, they did not specify the outcome of the patients diagnosed antenatally (28). In fact, the prognosis of fetal WT depends on several factors, including the presence of polyhydramnios, staging, histology type, and the presence of metastasis. Reports from Leclair et al. showed that 39% of fetal renal tumors had polyhydramnios as the main perinatal complication (23). Other reported complications include hypercalcemia, intra-tumoral hemorrhage, and non-immune hydrops fetalis leading to prematurity, fetal distress, and fetal demise (29). Our review of the data showed that the survival rate is excellent for infants who had isolated fetal WT, except for one patient who had disease progression secondary to therapeutic delays and suboptimal management leading to death (12) and another fetus that had several malformations that could be related to a syndrome and can explain the early mortality (14). On the other hand, all five patients who had fetal WT associated with polyhydramnios had a poor outcome and died in the early neonatal period. Moreover, polyhydramnios was responsible for fetal distress, hydrops, prematurity, and hemodynamic instability. Two patients died within 24 h of birth before the attempt of surgery. As for the remaining three patients, they were hemodynamically unstable, and when surgery was attempted to relieve the pressure from the tumor, it resulted in high morbidity, major postoperative complications, and death. In addition to kidney tumors, polyhydramnios has also been associated with other conditions, such as fetal gastrointestinal obstruction, neuromuscular disorders, fetal malformation syndromes, severe fetal anemia, maternal diabetes mellitus, macrosomia, and fetal hydrops and infection (TORCH), resulting in a poor neonatal outcome (30). Whether polyhydramnios was caused solely by WT or secondary to any other associated conditions could not be verified in this review and needs a larger study to investigate this hypothesis. Therefore, polyhydramnios is a worse prognostic factor associated with fetal WT. Special consideration should be directed to infants who are diagnosed with WT and polyhydramnios, as they need to be followed in a tertiary care center by an experienced multidisciplinary team.

Our case is illustrative of the management of a fetal WT. The fetal ultrasound detected a right renal mass at 32 WG without polyhydramnios nor fetal distress, and fetal MRI confirmed the presence of a solitary kidney mass without local dissemination. After the initial diagnosis, the parents were immediately referred to a tertiary care center, and the pregnancy was followed by a multidisciplinary team, including a specialized fetal radiologist, a pediatric nephrologist, a pediatric surgeon, and a neonatologist. Although the delivery could be done by the vaginal route, it was agreed with the parents to proceed with a CS because of the large size of the mass, thus decreasing the risk of tumor rupture. At birth, there were no documented episodes of hypercalcemia nor hypoglycemia. In addition, there was no family history, no dysmorphic features, or anomalies to suggest a predisposition syndrome. The infant underwent radical nephrectomy on the second day of life since she was hemodynamically stable. The pathologic examination of the mass showed a favorable histology WT, and confined to the renal capsule with negative lymph nodes, thus making it stage 1. These findings are similar to those in international studies where the majority of neonatal WT have favorable histology and low staging (28) and will not receive adjuvant chemotherapy. However, this infant had several concerning risk factors including a large tumor (approximately 500 g), unknown status of loss of heterozygosity (LOH) of 1p/16q, and the absence of a genetic study. After discussing the case in the multidisciplinary meeting, we upgraded this tumor to intermediate risk (19). Therefore, the female infant received dose-adjusted weekly vincristine over a period of 10 weeks according to the SIOP Umbrella RTSG 2016 protocol (24) without any complications.

What makes our case unique is the development after birth of isolated hemihypertrophy of the right lower limb, which can be associated with a predisposition syndrome (31). Hemihypertrophy is defined as an increase in length or circumference of one side of the body or limb compared to the contralateral part. It can be part of a syndrome, such as in BWS, or isolated, hence the new term ILO (32). On the other hand, BWS is a growth disorder commonly associated with WT. Manifestations of BWS can be detected during the fetal or neonatal period and include fetal macrosomia, macroglossia, polyhydramnios, placental mesenchymal dysplasia, and neonatal hypoglycemia (33). Our patient had no positive family history, no dysmorphic features, or other perinatal abnormal findings to suggest a diagnosis of BWS, making the diagnosis of ILO more likely. However, given the high index of suspicion, we continued screening for a potential predisposing syndrome until the development of hemihypertrophy. In a large review, MacFarland et al. reported that children with “syndromic” WT tend to be diagnosed earlier than “sporadic” WT, probably due to successful cancer screening programs. Furthermore, approximately 6% of children developed WT before the diagnosis of BWS/ILO by several months, with the youngest patient being 2 days old (34). In a prospective multicenter study of ILO, the prevalence of WT is approximately 3.5% (35), and in all reported cases, hemihypertrophy was present before the diagnosis of WT by at least 3 months (36). In our case report, the diagnosis of WT was made during the fetal period long before the development of the lower limb hemihypertrophy, an order of presentation that has not been reported before. The child benefitted, and continues to benefit, from a close screening program for early cancer detection since early infancy.

Early recognition of a predisposition syndrome is important not only to detect and promptly treat possible manifestations, but also to properly initiate surveillance programs. The benefits of screening for associated cancers (WT, hepatoblastomas, neuroblastomas) include early-stage detection of small and localized tumors, improved prognosis, less intensive treatment, and the omission of radiation therapy. Screening for non-cancerous conditions is equally essential for the early detection and management of potential morbidities, namely, hypoglycemia/hyperinsulinism and kidney and musculoskeletal disorders. Genetic testing remains essential in confirming the diagnosis and for genetic counseling.

## Conclusion

This case illustrates the challenging management of fetal WT. Prenatal ultrasound remains an essential tool for diagnosis. Immediate care should be offered in a tertiary care center by an experienced multidisciplinary team. Surgical resection is the standard of care after stabilization of the newborn. The prognosis is generally excellent but depends on the tumor's staging, histology, and on the presence of polyhydramnios, which is a worse prognostic factor. Fetal WT can be associated with predisposition syndromes, but their manifestations may develop after the diagnosis of cancer has been made. Physicians should be aware of this possibility and not be satisfied with treating only the tumor. Therefore, they are encouraged to continue screening for predisposition syndromes and to offer genetic testing to start surveillance programs and plan early interventions.

## Data Availability

The raw data supporting the conclusions of this article will be made available by the authors, without undue reservation.
